# Pyelo‐Hepatic Fistula Resulting From a Perinephric Abscess: A Case Report and Literature Review

**DOI:** 10.1155/criu/2556392

**Published:** 2026-05-20

**Authors:** Jonathan Alcantar, Emily Hahn, Anthony Castelvecchi, Mark Wille

**Affiliations:** ^1^ Division of Urology, Department of Surgery John H. Stroger, Jr. Hospital of Cook County, Chicago, Illinois, USA, cookcountyhhs.org

**Keywords:** liver abscess, nephrolithiasis, perinephric abscess, *Proteus mirabilis*, pyelo-hepatic fistula

## Abstract

The extension of a renal infection to the liver is extremely rare. We present the case of a 70‐year‐old woman with right hip pain and delayed detection of a perinephric abscess involving her liver. Tomographic and endoscopic contrast studies revealed the presence of a fistulous tract between her liver and right kidney. With minimally invasive intervention, including placement of a ureteral stent, two hepatic drains, a nephrostomy tube, and culture‐directed antimicrobial therapy, her infection resolved. Closure of the fistula was subsequently confirmed radiographically. This is the first reported incidence of a pyelo‐hepatic fistula resulting from a perinephric abscess.

Keynote Message

A pyelo‐hepatic fistula is a rare complication that occurs from extension of a renal infection or primary malignancy to the liver. We present a case of a woman with an obstructing renal stone found to have a perinephric abscess that progressed to a pyelo‐hepatic fistula. Complete resolution of her infection and fistula was achieved through minimally invasive measures, including antibiotics and percutaneous drains.

## 1. Introduction

A perinephric abscess is a localized collection of purulence located beyond the renal capsule but contained within Gerota′s fascia. It typically arises from the extravasation of infected urine due to obstruction, rupture of an acute cortical abscess into the perinephric space, or hematogenous seeding from a distant infection. Diagnosing perinephric abscesses can be challenging, as the onset of symptoms is often insidious and can mimic pyelonephritis. If left untreated, the infection can track beyond Gerota′s fascia to involve adjacent anatomical structures, most commonly neighboring intestinal segments, lungs, and the psoas muscle [1]. Extension of a renal infectious focus to the liver is extremely rare, with only five cases reported in the literature [2–6]. This report describes a previously unreported mechanism of pyelo‐hepatic fistula formation resulting from an extension of a perinephric abscess associated with an obstructing renal calculus. Unlike previously described cases caused by chronic infiltrative disease such as xanthogranulomatous pyelonephritis (XGP) or renal malignancy, the fistula in this case developed through acute suppurative inflammation and was successfully managed with minimally invasive interventions.

## 2. Case Report

A 70‐year‐old woman with poorly controlled diabetes and atrial flutter on clopidogrel presented to our hospital as a direct transfer from an outside facility where she had been admitted for 6 weeks. She initially presented with intermittent right hip pain. On presentation, her temperature was 98.6°F, blood pressure 189/67 mmHg, and pulse 91 beats/min. Serum studies revealed a white blood cell count of 12.8 k/*μ*L, creatinine 1.38 mg/dL, and serum glucose 519 mg/dL. An abdominal ultrasound identified a 3.6‐cm hypoechoic lesion in the right hepatic lobe and a 3.4‐cm simple‐appearing cyst in the upper pole of the right kidney.

**Figure 1:**
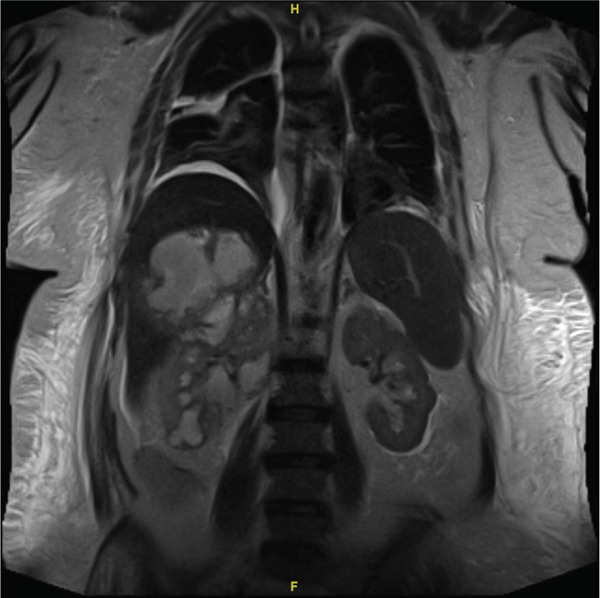
Magnetic resonance imaging performed 7 days after initial presentation demonstrates right hydronephrosis secondary to an obstructing ureteropelvic junction (UPJ) stone. A large, complex intrarenal abscess is present in the upper pole of the right kidney, measuring 5.7 × 5.9 × 6.2 cm, with extension into the perinephric space and direct abutment of a second multiloculated collection in the right hepatic lobe measuring 7 × 7.6 × 6 cm. These findings raise concern for extension of the perinephric abscess into the liver; however, a definitive fistulous tract is not visualized on this study.

**Figure 2:**
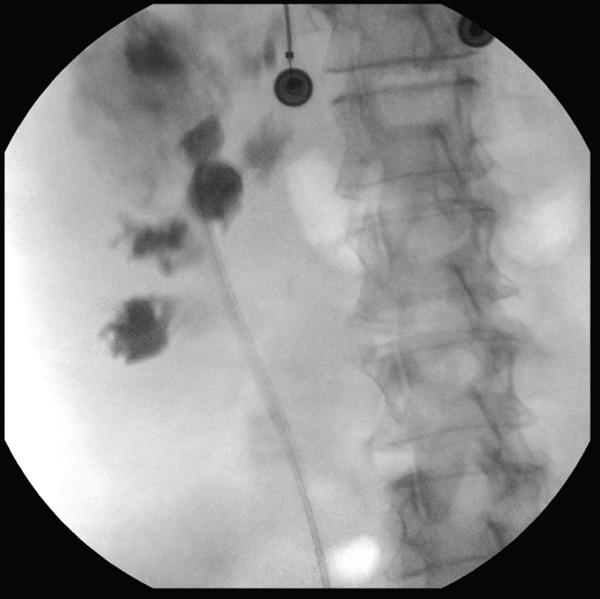
Retrograde pyelogram performed intraoperatively during urgent right ureteral stent placement demonstrates contrast extravasation just superior and lateral to the upper pole of the kidney, suggesting a contained leak. The pattern of contrast distribution suggests a communication between the renal collecting system and the adjacent liver, supporting the presence of a pyelo‐hepatic fistula.

Magnetic resonance imaging (MRI) performed 7 days later (Figure 1) revealed a 2.5‐cm obstructing right renal stone at the Ureteropelvic Junction with upstream hydronephrosis, along with two complex multiloculated fluid collections: a 7.6‐cm T2‐signal hyperintense collection in the posterior right hepatic lobe, and a 6.2‐cm partially intrarenal and perirenal abscess originating from the superior pole of the right kidney and abutting the liver. A right ureteral stent was urgently placed, and intraoperative retrograde pyelogram (Figure 2) showed contrast extravasation contained superiorly and laterally to the collecting system. Although these findings were suspicious for a possible communication between the renal collecting system and the hepatic fluid collection, no definitive fistulous tract was visualized at that time.

**Figure 3:**
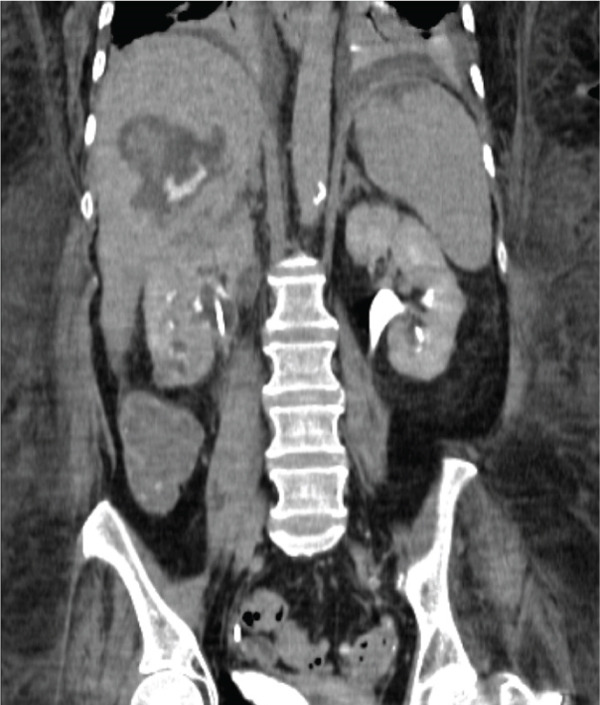
Computed tomography (CT) urogram performed 20 days after original presentation shows contrast extravasating from the right kidney and reaching the right hepatic lobe, confirming a pyelo‐hepatic fistulous tract.

Given the persistent concern for a possible communication between the collecting system and the hepatic fluid collection, a computed tomographic (CT) urogram was obtained to evaluate for contrast extravasation from the renal collecting system (Figure 3). At the time of imaging, the patient′s renal function had improved, with a serum creatinine of 0.95 mg/dL and an estimated glomerular filtration rate of approximately 51 mL/min/1.73 m^2^, matching her baseline renal function. The CT urogram demonstrated persistent right hydronephrosis and contrast extravasation from the superior pole of the right kidney extending into the right hepatic lobe, confirming the presence of a pyelo‐hepatic fistulous tract.

After holding all anticoagulation for 5 days, a drain was placed into the hepatic fluid collection under CT guidance, which required replacement 7 days later to improve drainage. A total of 100 mL of purulent material was aspirated from the two drains, and cultures grew multidrug‐resistant *Proteus mirabilis*. The patient was treated with intravenous meropenem and linezolid, followed by oral trimethoprim–sulfamethoxazole and metronidazole, for a total of 28 days of antibiotics.

Over the 2 weeks following hepatic drain placement, the patient′s renal function progressively declined, prompting transfer to John H. Stroger Hospital for escalation of care. On arrival, her serum creatinine was 3.3 mg/dL, and her glomerular filtration rate was 14 mL/min/1.73 m^2^. A CT scan showed persistent right hydronephrosis but resolution of the perinephric and liver abscesses. A right nephrostomy tube was placed, and her renal function subsequently stabilized. The second hepatic drain was removed 22 days after its placement, the day before her discharge.

**Figure 4:**
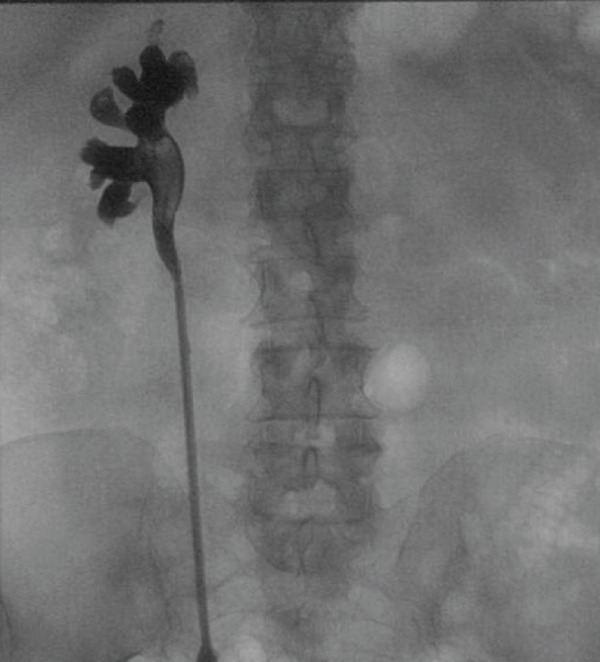
Retrograde pyelogram was obtained during definitive ureteroscopic stone removal, performed several weeks after removal of the patient′s hepatic drains. The radiograph shows no contrast extravasation, indicating complete resolution of the pyelo‐hepatic fistula. A filling defect in the ureteropelvic junction corresponds to the 2.5‐cm stone.

The patient returned for outpatient ureteroscopic treatment of her right ureteral stone. Intraoperatively, no contrast extravasation was observed on retrograde pyelogram (Figure 4), confirming the resolution of the pyelo‐hepatic fistulous tract. The brown matrix stone was treated and confirmed to be composed of carbonate apatite on stone analysis.

## 3. Discussion

Perinephric abscesses are an uncommon complication of upper urinary tract infections, with a reported incidence ranging from 1 to 10 cases per 10,000 hospital admissions [1]. Immunocompromised patients, particularly those with diabetes, are at increased risk; up to one‐third of patients who develop a perinephric abscess are diabetic [7]. *E. coli* and *P. mirabilis* are the most common causative organisms when the primary source of infection is the urinary tract, whereas *Staphylococcus aureus* is usually detected when the perinephric abscess results from hematogenous dissemination from a distant site. In younger patients presenting acutely, the source of infection is often an abscess within the renal parenchyma that ruptures into the perinephric space. However, this is less common than the chronic form of perinephric abscess, which typically results from an upper tract infection in the setting of an obstructing renal calculus. [1] The synergistic effects of high intrapelvic pressure and bacterial overgrowth due to urinary stasis upstream of the obstructing stone can have a destructive effect on the kidney. This can lead to disruption of renal parenchyma, allowing the extravasation of infected material into the perinephric space [1]. Alternatively, if the infected urine remains confined within the kidney and is not adequately treated, it can progress to XGP, characterized by diffuse destruction of renal parenchyma.

This patient′s multiple medical comorbidities, particularly her poorly controlled diabetes, likely contributed to the advanced progression of her disease. Diabetes is a well‐established risk factor for the development of perinephric abscesses from upper genitourinary tract infections, with diabetic patients accounting for nearly two‐thirds of cases and exhibiting a nearly fourfold increased risk of this complication [8, 9]. Several mechanisms have been proposed to explain this association. Glucosuria can increase bacterial adherence to uroepithelial cells and create a nutrient‐rich environment that fosters the proliferation of uropathogens [10, 11]. Additionally, the neutrophil and macrophage dysfunction that is observed in patients with hyperglycemia may compromise host immune function and antibacterial activity [12, 13]. These factors collectively establish a microenvironment that promotes bacterial overgrowth while compromising the host′s ability to detect and mount an effective immune response, thereby increasing the risk of significant complications from an upper urinary tract infection.

The acute renal failure (ARF) this patient developed, in the setting of Stage IIIa chronic kidney disease (CKD), was likely multifactorial. Impaired renal autoregulation, vascular dysfunction, and reduced nephron reserve in CKD increase susceptibility to additional insults such as infection‐related hypotension, obstructive uropathy, and intravenous contrast load [14, 15]. Clinicians should maintain a heightened awareness of the increased risk of renal deterioration in this patient population. CT urography ultimately provided definitive confirmation of the fistulous tract by demonstrating contrast extravasation from the collecting system into the hepatic parenchyma. Although contrast exposure may pose a risk in patients with impaired renal function, the patient′s renal function had improved at the time of imaging. In similar scenarios, alternative approaches such as contrast injection through a percutaneous drain or nephrostomy tube may help demonstrate fistulous communication while minimizing additional nephrotoxic exposure.

By definition, a perinephric abscess is located beyond the renal capsule but contained within Gerota′s fascia. The reflection and fusion of Gerota′s fascia with other fascial entities provides an anatomical barrier to the spread of infection. However, contiguous spread to adjacent structures is possible. The peritoneum is the most common site for perinephric abscess extension, with reports of this infectious entity spreading to intestinal segments, resulting in reno‐duodenal and reno‐colonic fistulas [16, 17]. Other possible sites of spread include the retroperitoneum, psoas muscle, vena cava, renal vein, and pleural cavity.

Perinephric abscess management depends on the size and chronicity of the abscess [1]. Smaller abscesses may be treated conservatively with antibiotics and correction of predisposing factors, such as diabetes or urinary tract obstruction. Larger or loculated abscesses often require percutaneous drainage, with possible drain repositioning if inadequate decompression occurs. In refractory cases or when significant anatomical disruption is present, surgical intervention may be necessary.

Extension of renal infection to the liver is extremely rare, with only five cases reported in the literature [2–6]. Most previously reported cases describe hepatic abscess formation from contiguous spread of renal infection rather than a true fistulous communication between the renal collecting system and hepatic parenchyma.

An even less commonly observed entity is the pyelo‐hepatic fistula, with only two prior cases reported [4, 18]. Chung et al. reported the first case of a pyelo‐hepatic fistula secondary to XGP, in which imaging demonstrated a severely diseased right kidney with multiple cavities extending through the perinephric space into the right hepatic lobe [4]. The patient required right nephrectomy and partial hepatectomy, with pathology confirming XGP and fistulous communication. Varshney et al. reported a second case resulting from direct invasion of the liver from renal squamous cell carcinoma in the setting of a nonfunctioning kidney with an obstructing staghorn calculus [18]. This patient also required surgical management with nephrectomy, and pathology confirmed malignant extension as the mechanism of fistula formation.

In all three cases, fistula formation resulted from direct extension of a destructive pathologic process facilitated by the close proximity of the superior pole of the right kidney to the liver. However, the mechanism in our case differs from those previously reported. In the prior cases, fistula formation occurred in the setting of chronic infiltrative disease, either diffuse granulomatous inflammation from XGP or direct malignant invasion, both of which ultimately required nephrectomy. In contrast, our case suggests that a perinephric abscess can serve as the primary driver of fistula formation through acute suppurative inflammation and pressure‐related tissue breakdown. The synergistic effects of urinary obstruction, elevated intrapelvic pressure, and bacterial overgrowth can disrupt renal parenchyma and allow infected material to extend into the perinephric space and adjacent hepatic tissue. This represents a distinct and previously unreported mechanism of pyelo‐hepatic fistula formation compared with extension from XGP or malignancy.

This case represents the first instance of a pyelo‐hepatic fistula likely resulting from a perinephric abscess and illustrates how it was effectively treated with minimally invasive approaches, leading to complete resolution. It highlights the importance of maintaining a high index of suspicion for complex infectious processes in immunocompromised patients, particularly those with diabetes, who may present with atypical symptoms, potentially delaying diagnosis and allowing progression to severe complications. Early identification and intervention are crucial in achieving favorable outcomes.

## 4. Conclusion

This case illustrates a previously unreported mechanism of pyelo‐hepatic fistula formation resulting from extension of a perinephric abscess associated with an obstructing renal calculus. Multimodal imaging—including retrograde pyelography and CT urography—was required to establish the diagnosis. Unlike previously reported cases associated with XGP or renal malignancy, this fistula resolved with minimally invasive management consisting of urinary diversion, percutaneous drainage, and targeted antimicrobial therapy.

## Funding

No funding was received for this manuscript.

## Consent

Written informed consent was obtained from the patient for publication of this case report and accompanying images.

## Conflicts of Interest

The authors declare no conflicts of interest.

## Data Availability

The data that support the findings of this study are available on request from the corresponding author. The data are not publicly available due to privacy or ethical restrictions.
